# Assessing the Influence of Water Management and Rainfall Seasonality on Water Quality and Intestinal Parasitism in Rural Northeastern Brazil

**DOI:** 10.1155/2018/8159354

**Published:** 2018-07-18

**Authors:** Alexandre Pessoa Dias, Deiviane Calegar, Filipe Anibal Carvalho-Costa, Maria de Fátima Leal Alencar, Caroline Ferraz Ignacio, Milena Enderson Chagas da Silva, Antonio Henrique Almeida de Moraes Neto

**Affiliations:** ^1^Fundação Oswaldo Cruz, Instituto Oswaldo Cruz, Laboratório de Inovações em Terapias, Ensino e Bioprodutos (LITEB), Rio de Janeiro, Brazil; ^2^Fundação Oswaldo Cruz, Escola Politécnica de Saúde Joaquim Venâncio, Laboratório de Educação Profissional em Vigilância em Saúde (LAVSA), Rio de Janeiro, Brazil; ^3^Fundação Oswaldo Cruz, Instituto Oswaldo Cruz, Laboratório de Epidemiologia e Sistemática Molecular (LESM), Rio de Janeiro, Brazil; ^4^Escritório Técnico Regional Fiocruz Piauí, Brazil

## Abstract

**Introduction:**

The drought in the Brazilian semiarid region has affected the quality of water. This study assessed the relationships between enteric parasitoses, water management, and water quality, correlating them with pluviometric seasonality.

**Methods:**

Cross-sectional surveys were carried out in four rural communities at the beginning of the dry season (n=151), at the end of the dry season (n=184), and in the rainy season (n=199), in order to collect sociodemographic data, human fecal samples, and samples of the water used for human consumption for physicochemical and microbiological analyses. In 2015, water filters were provided to 30 households under study.

**Results:**

There was an increasing trend in detection rates of commensal protozoa and the* Entamoeba histolytica/Entamoeba dispar* complex at the beginning of the rainy season, with detection rates of 6% in 2014 and 21.6% in 2016.* Giardia intestinalis* and* Ascaris lumbricoides *presented distinct temporal distributions, which peaked in 2015: 20.1% and 30%, respectively. The proportion of inhabitants drinking inadequate water was 55% at the beginning of the dry season and 28.8% at the end of the dry season, reaching 70.9% at the beginning of the rainy season. The presence of filters reduced this proportion among those who received the hollow ceramic candle filter.

**Conclusions:**

Data suggest that the strategies to increase water supply in the Brazilian semiarid region can be ameliorated in order to improve the quality of drinking water.

## 1. Introduction

The World Health Organization (WHO) proposes that low-cost interventions for water treatment and safe storage in households bring significant improvements in water quality and, consequently, in the control of waterborne and diarrheal diseases [1, 2]. The Brazilian semiarid region, located mainly in the Northeast region, covers 982,563.3 km^2^, nine states, and 1,133 municipalities with a population of 22.5 million inhabitants [3–5]. The Northeast region accounts for almost 60% of the country's 16.3 million inhabitants currently living in extreme poverty; 56.4% live in rural areas [6] where only 33.4% of the households are connected to the water supply network and 5.1% to the sewage collection network [7].

In order to increase the water supply in the semiarid region, governmental initiatives have adopted rainwater harvesting in an attempt to utilize the strong pluviometric seasonality of the region, which is characterized by a rainy season concentrated in four months of the year [8]. In 2015, Brazil experienced the prolongation of one of the most severe droughts of the last 50 years, with its most critical period being between 2012 and 2014 [9]. In Brazil, the federal government's Water for All (*Água para Todos*) program has expanded the implementation of rainwater harvesting tanks, in accordance with guidelines of the Brazil without Extreme Poverty Plan [10, 11]. This program reached its target for the 2011-2014 period supplying 750,000 families in the Brazilian semiarid region. These families had limited access to drinkable water and depended on water sources that were unreliable or inappropriate for consumption [12]. According to data from the Ministry of Social and Agrarian Development, 1,257,670 cisterns were built in the semiarid region from 2003 to 2017 to harvest rainwater for human consumption [13]. In the case of the state of Ceará, there were 289,338 units for the same period. The cisterns are built with cement or polyethylene, have the capacity to store 16 m^3^ of rainwater, and are connected to the roofs of houses. During the rainy season, the rainwater harvested from the roof accumulates in the cistern. When it is full, the tank provides water for a family of up to five people for a period of up to eight months, which corresponds to a per capita consumption of about 14L/inhabitant/day [14].

Microbiological analyses of the cisterns' harvested rainwater point to the presence of contamination with coliforms in South Africa [15] and northeastern Brazil [16, 17]. On the other hand, research shows that the presence of cisterns represents a protection factor against diarrhea and intestinal parasites [18, 19]. Therefore, water management must accompany the scaling up of the implementation of rainwater cisterns in order to achieve the control of waterborne diseases. It is possible that in addition to the greater supply of water through the use of cisterns improvements can be made to water quality. The present study aims to assess the correlation between human infection with intestinal parasites, water management, and water quality, correlating them with pluviometric seasonality, in four rural communities of a settlement in the Brazilian semiarid region.

## 2. Population and Methods

### 2.1. Description of the Study Area

The study was carried in the municipalities of Madalena and Quixeramobim of the State of Ceará, in four rural communities (São Joaquim Sede, São Joaquim Raiz, Quieto 1, and Quieto 2) with a total population of 455 inhabitants, belonging to the Settlement 25 de Maio (latitude: 5° 1′40.52 ′′S; longitude: 39° 31′54.85°) with an area of 22,992 ha [20, 21]. The region is characterized as being hot tropical semiarid (Köppen classification), with a dry and hot period from June to January with extremely low rainfall and a rainy period in February to May [22]. The average annual rainfall between 2012 and 2016 ranged from 299.2 to 461.9 mm per year. Considering that the historical average of the rainy season oscillates around 606 mm, it was observed that, during the study period, there was an annual rainfall deficit between 24% and 51%, illustrating the additional water stress to which the analyzed population was subjected.

### 2.2. Study Design

Cross-sectional surveys were carried out: (i) at the beginning of the dry season (July/August 2014 [n=40 houses; 151 subjects]), (ii) at the end of the dry season (October 2015 [n=60 houses; 184 subjects]), and (iii) in the rainy season (April/May 2016 [n=59 houses; 199 subjects]). Researchers collected sociodemographic data, fecal samples of the residents, and samples of the water used for human consumption. In 2015 water filters were provided to 30 (50%) of households under study. Among the filters distributed, 15 had a traditional porous candle (hollow, without charcoal), classified as P-III (Brazilian Association of Technical Standards, Portuguese acronym ABNT) [23] with a particle retention range between 5 and 15 *μ*m, and 15 had porous candles (with charcoal), classified as P-I with a retention range between 0.5 and 1 *μ*m (ABNT) [23]. Data were analyzed using the Epi-Info 7.0 Program, using chi-square or Fisher's exact test for comparison of proportions. The level of statistical significance was set at p <0.05.

### 2.3. Parasitological Analyses of Feces

Fecal samples were collected from the residents in wide-mouth screw-cap containers with scoops, without preservatives. The samples were analyzed by the Lutz [24] technique (spontaneous sedimentation), with a reading of three slides per sample, stained by Lugol. The same experienced technician examined all samples. The parasitized individuals received treatment at home visits, under medical supervision of the local primary health unit of the respective municipalities, during the three campaigns.

### 2.4. Physical-Chemical and Microbiological Analyses of Water

From each household (except two in 2016) a 200 mL and 250 mL water sample was collected in a sterile flask (with sodium thiosulphate), for physicochemical and microbiological analysis, respectively. Samples were collected from the residential source, at a time-point directly before consumption by the inhabitants. The sources of water collected at the most recent point before consumption were varied, including rainwater, wells, ponds or bottled water, or water from the neighbors' house. The* in situ *measurements to determine the water sample pH, electrical conductivity, dissolved oxygen, total dissolved solids, salinity, and temperature were performed using the Hanna® Instruments (São Paulo, Brazil) model HI9828 multiparameter probe. The analysis of water salinity followed that prescribed in National Council for the Environment's Resolution no. 357 (Conama 2005) [25], which classifies the waters into fresh water (salinity up to 0.05%), brackish water (salinity from 0.05% to 3%), and salt water (salinity above 3%). To determine the physicochemical parameters turbidity and color and the microbiological parameters total coliforms and* Escherichia coli*, the water samples were sent with a maximum period of 24 hours to the Central Health Laboratory of Ceará (LACEN-CE), according to the collection procedures prescribed in the Manual for the Collection, Packaging, and Transport of Samples (Ceará 2013) and according to NBR ISO/IEC 17,025 (ABNT 2005). Water classification (adequate or inadequate), considering microbiological criteria, was based on the presence of* Escherichia coli*. Total coliform was not a criterion for classifying water as inadequate, since this includes environmental microorganisms not necessarily indicative of fecal contamination. Considering physicochemical criteria, water was classified as adequate or inadequate according to standards described in Administrative Rule no. 2914 of the Brazilian Ministry of Health [26]. The following techniques were performed in LACEN-CE: (i) turbidity: nephelometric method, 2130B, APHA 2012 [27]), (ii) apparent color (visual comparison method, 2120B, APHA 2012 [27]), and (iii)* Escherichia coli *and total coliforms: chromogenic/enzymatic substrate technique (9223B APHA 2012 [27]).

## 3. Results

### 3.1. Prevalence of Intestinal Parasite Infections in Different Climatic Seasons and Years

**Figure 1:**
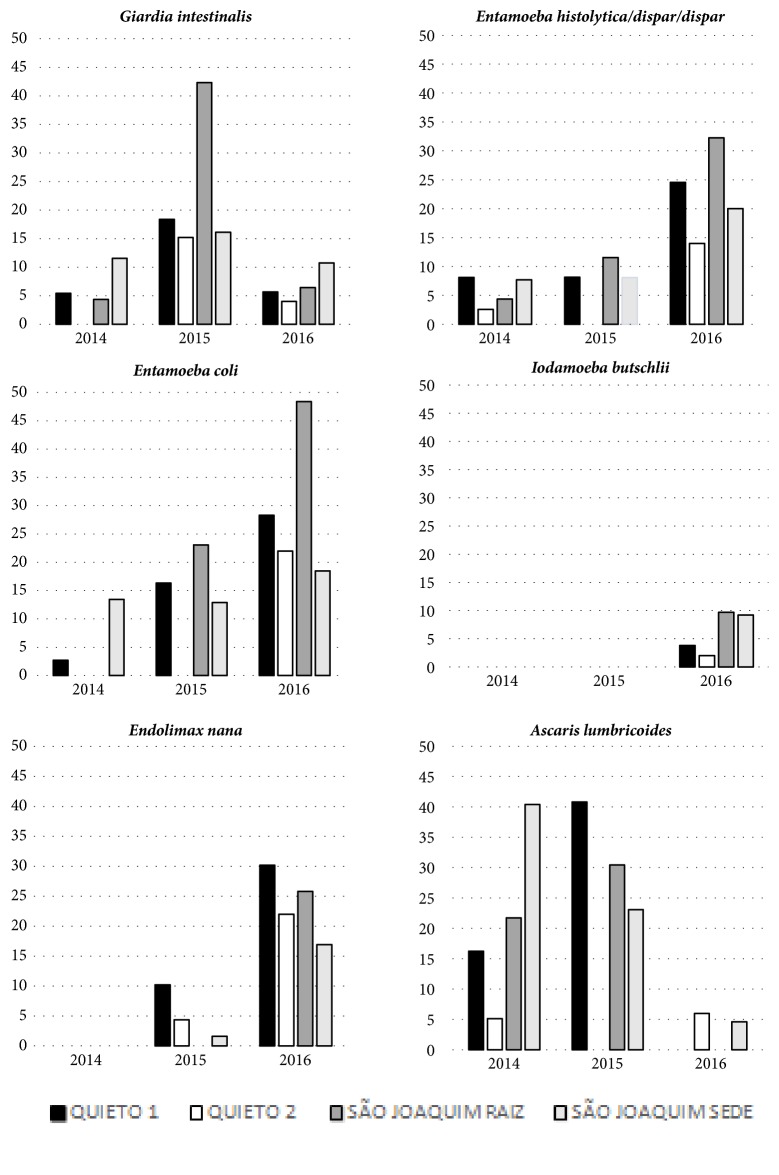
Prevalence of intestinal parasitoses in the communities Quieto 1, Quieto 2, São Joaquim, Sede, and São Joaquim, Raiz, of Assentamento 25 de Maio, Ceará, in the years 2014, 2015, and 2016.

**Figure 2:**
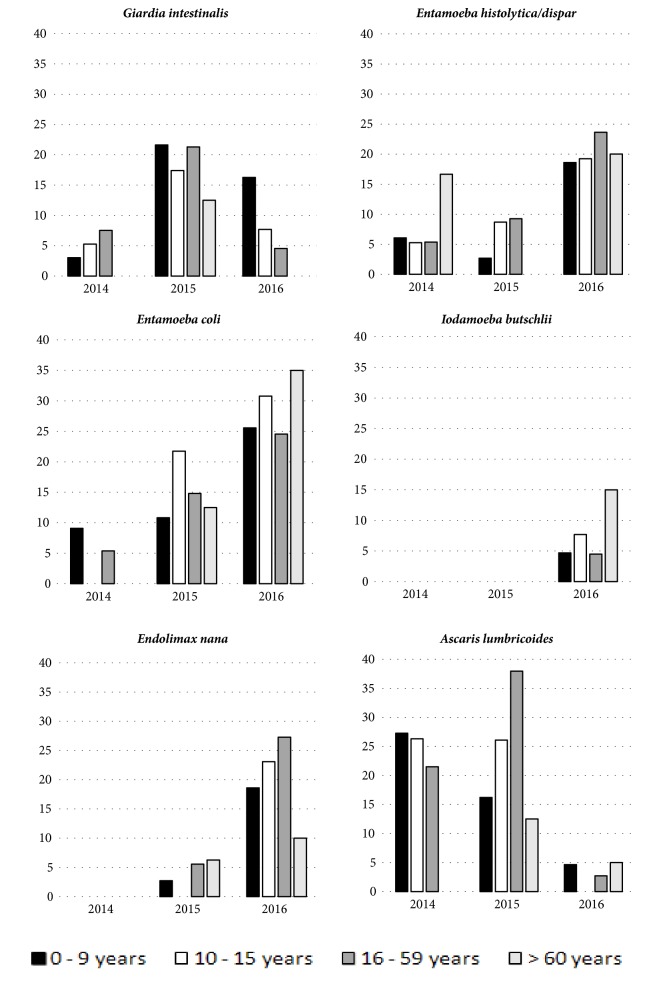
Prevalence of intestinal parasitoses by age group in Assentamento 25 de Maio, Ceará, in the years 2014, 2015, and 2016.

Table 1 shows the frequency of detection of different intestinal parasites in relation to sociodemographic characteristics. In 2014 households of 3-5 residents exhibited the highest prevalence of intestinal parasites. In 2015 all (7/7) species were found to be most prevalent in houses with more than 5 individuals and in 2016 this was true for 5 out of 7 species. As shown in Figures 1 and 2, there was an increase in detection rates of commensal protozoa and the* Entamoeba histolytica/Entamoeba dispar *complex in the year 2016, which corresponded to the beginning of the rainy season. In this fashion the detection rate of* E. histolytica/E. dispar *increased from 6.0% (9/151) in 2014 to 21.6% (43/199) in 2016. With respect to the commensal protozoa* Entamoeba coli*, there was an increase from 5.3% (8/151) in 2014 to 26.6% (53/199) in 2016.* Giardia intestinalis *presented a distinct temporal distribution and together with* Ascaris lumbricoides *was detected more frequently in 2015 (20.1% [37/184] and 30% [55/184], respectively), with a reduction in 2016 (7.0% [14/199] and 3.0% [6/199], respectively). The São Joaquim Raiz community tended to present higher prevalence rates for intestinal parasites in 2015 and 2016 (Figure 1). In relation to age groups,* G. intestinalis *infection tended to be more frequent in children up to nine years of age in the years 2015 and 2016, with prevalence rates reaching 21.6% and 16.3%, respectively, in this age group. In 2014, prevalence of giardiasis fluctuated between 3% and 7.5% in distinct age groups. Considering* E. histolytica*/*E. dispar*, detection rates were higher in subjects aged 16 to 59 years, reaching 9.3% and 23.6% in the years 2015 and 2016, respectively. In 2014, prevalence with* E. histolytica*/*E. dispar *reached 16.7% among individuals aged > 60 years. Ascariasis was present in all age groups, with detection rates from 21.5% to 27.3% in different age groups in 2014 and from 16.2% to 38% in 2015 (Figure 2).

### 3.2. Water Quality and Its Association with Infection by Intestinal Parasites

The proportion of inhabitants drinking inadequate water was higher at the beginning of the dry season (2014) (55.0% [83/151]) and at the beginning of the rainy season (2016), reaching 70.9% (141/199). In 2015, at the end of the dry season, 28.8% (53/184) of the studied subjects were consuming inadequate water (not shown). As observed in Table 2, there was no significant difference in the presence of different intestinal parasites in relation to the drinking water quality classified as satisfactory or unsatisfactory. In the three studied years, parasites such as* G. intestinalis *and* E. histolytica/E. dispar *were detected with similar frequencies among subjects drinking satisfactory or unsatisfactory water. Interestingly, in 2014,* A. lumbricoides *and* Entamoeba coli *were more frequently detected in subjects drinking satisfactory water.

### 3.3. Impact of Filters on the Prevalence of Intestinal Parasites and on Water Quality

As shown in Table 3, both types of water filters distributed in 2015 did not affect the prevalence of different intestinal parasites in 2016. Nevertheless, filters influenced the microbiological quality of water. In 2016, the proportion of individuals consuming unsatisfactory water was 57.1% (48/84) among those who received the hollow ceramic candle filter, 63.2% (43/68) among those who received the ceramic candle filter with charcoal, 77.4% (24/31) among those who already had a filter, and 95.1% (78/82) among those who did not use any type of filter at home (p<0.001). There was no statistically significant difference between the proportions of households with unsatisfactory water between the two types of ceramic candles used in household filters.

## 4. Discussion

This study shows a higher presence of infection with intestinal protozoa when compared to soil-transmitted helminthes, in accordance with a current trend of decrease in the prevalence of the latter [28]. Although some protozoa are not pathogenic, they can be considered indicators of water quality, since they are waterborne organisms transmitted by the fecal-oral route.

Mass drug administration in Brazil prioritizes the use of anthelmintic drugs (single 400 mg oral Albendazole dose), in the context of the integrated plan of action for the elimination of neglected diseases [29]. Consonant with the recommendations of WHO, these strategies do not include intestinal protozoa, which can lead to an increase in the prevalence of these organisms [30]. In the studied region, treatment for soil-transmitted helminthes is performed by the primary healthcare system.

In the case of the* E. histolytica/E. dispar *complex it is necessary to carry out specific diagnostic tests to differentiate them. Estimates indicate that* E. dispar *is about ten times more frequent than* E. histolytica *and with a wide geographic distribution [31]. A study carried out in Ceará demonstrated that* E. dispar *is more frequent than* E. histolytica *in a scenario of drought [32]. Infection with* G. intestinalis *is also common in the studied localities. Studies have demonstrated that chronic and apparently asymptomatic infection with* G. intestinalis *negatively affects the nutritional status of children in developing countries [33]. This study demonstrated that prevalence of waterborne commensal protozoa (*Endolimax nana*,* Iodamoeba butschlii, *and* Entamoeba coli*) and* E. histolytica/E. dispar *tends to increase in the rainy season, when water is more abundant and the harvesting of rainwater begins. This coincides with a higher proportion of dwellers ingesting inadequate water. The prolongation of the drought in the studied period has resulted in the search for different sources of water for consumption, including the water transported by truck from dams and rivers distant from the settlement. The higher occurrence of unsatisfactory drinking water from households in the rainy season suggests that there is a greater contamination of water supply and, therefore, a correlation between the beginning of the rainy season and the increase in the prevalence of intestinal parasites. Early rainwater promotes the cleaning of roofs and water margins, resulting in the transport of pathogens into natural reservoirs and cisterns.

Data suggests an ecological association, at the community level, between the increase in prevalence of some species of enteric protozoa and the decrease of water quality at the beginning of the rainy season. Nevertheless, at the individual level such an association could not be demonstrated, since rates were similar among inhabitants drinking microbiologically adequate or inadequate water. This lack of association between the microbiological quality of water and infection by intestinal parasites (including a higher positivity for* A. lumbricoides *and* Entamoeba coli *in people who drink water of satisfactory quality) was an interesting finding of this work. This suggests a dissociation, at the domiciliary level, between the microbiological quality of water and the transmission of intestinal parasites. This transmission, therefore, should have more complex dynamics and involve other aspects and determinants, independents of the bacterial contamination of the water and involving the domestic, peridomestic, and working (agricultural) environment. Lack of association at the individual level indicates the possibility of water consumption in several households beyond that actually inhabited by the subject. In addition, water quality analyses do not consider the presence of infectious forms of intestinal parasites, so that, theoretically, microbiologically satisfactory water could be contaminated with intestinal parasitic cysts and eggs. This possibility is supported by the fact that these structures are less sensitive to water chlorination.

Although the protective effect of the filters on intestinal parasites was not observed, a significantly lower proportion of inadequate water consumption was observed among the families that received the filters. This shows that the filters have a positive impact on the quality of the water to be consumed and can influence the transmission of bacterial waterborne diseases. Previous studies indicate the efficiency of the filter in the removal of bacteria such as* Escherichia coli *and total coliforms from drinking water in rural Bolivia and South Africa [34, 35].

## 5. Conclusions

The data suggest that the strategies to increase water supply in the Brazilian semiarid region can include improvements in the quality of rainwater harvested for human consumption. In this sense, the distribution of household-based filters can be considered an effective solution when used in conjunction with rainwater harvesting systems and chlorination, which should be made available to low-income rural inhabitants. Periodic assessments of the water quality of cisterns should be conducted, with emphasis on microbiological aspects. Also, water supply expansion programs in Brazil should incorporate household water treatment with health education to serve as a sanitation barrier for the control of intestinal parasitic infections.

## Figures and Tables

**Table 1:** Prevalence of intestinal parasitic infections in relation to socioeconomic data: household occupancy rate, per capita monthly income range, and presence of toilet in the years 2014, 2015, and 2016 in the rural communities studied in Settlement 25 de Maio, Ceará State, Brazil.

**Year**	***E. nana***	***I.*** ***Butschlii* ** **(**%**)**	***E. coli* ** **(**%**)**	***E.*** ***histolytica*** ***/ dispar*** **(**%**)**	***G.*** ***intestinalis* ** **(**%**)**	***A.*** ***lumbricoides* ** **(**%**)**	***H. nana* ** **(**%**)**
**2014 (*n*=151)**							

**Household occupancy rate**							
1-3	-	-	6.45 (2/31)	6.45 (2/31)	3.23 (1/31)	32.26 (10/31)	0 (0/31)
3-5	-	-	7.14 (4/56)	7.14 (4/56)	10.71 (6/56)	23.21 (13/56)	1.79 (1/56)
>5	-	-	3.13 (2/64)	4.69 (3/64)	3.13 (2/64)	17.19 (11/64)	0 (0/64)
**Range of monthly income per capita** ^**1**^							
0-44	-	-	4.26 (4/94)	4.26 (4/94)	6.38 (6/94)	17.02 (16/94)	0 (0/94)
45-88		-	6.25 (2/32)	6.25 (2/32)	3.13 (1/32)	25.00 (8/32)	3.13 (1/32)
>88	-	-	8.00 (2/25)	12.00 (3/25)	8.00 (2/25)	40.00 (10/25)	0 (0/25)
**Presence of toilet**							
yes	-	-	6.35 (8/126)	6.35 (8/126)	6.35 (8/126)	22.22 (28/126)	0.79 (1/126)
no	-	-	0 (0/25)	4.00 (1/25)	4.00 (1/25)	24.00 (6/25)	0 (0/25)

**2015 (*n*=184)**							

**Household occupancy rate**							
1-3	2.13 (1/47)	-	8.51 (4/47)	8.51 (4/47)	14.89 (7/47)	36.17 (17/47)	-
3-5	2.82 (2/71)	-	15.49 (11/71)	2.82 (2/71)	9.86 (7/71)	18.31 (13/71)	-
>5	7.58 (5/66)	-	18.18 (12/66)	10.61 (7/66)	34.85 (23/66)	39.39 (26/66)	-
**Range of monthly income per capita** ^**1**^							
0-44	5.88 (6/102)	-	15.69 (16/102)	6.86 (7/102)	25.49 (26/102)	38.24 (39/102)	-
45-88	0 (0/44)	-	13.64 (6/44)	11.36 (5/44)	20.45 (9/44)	15.91 (7/44)	-
>88	5.26 (2/38)	-	13.16 (5/38)	2.63 (1/38)	0 (0/38)	5.26 (2/38)	-
**Presence of toilet**							
yes	4.55 (7/154)	-	15.58 (24/154)	5.84 (9/154)	18.83 (29/154)	32.47 (50/154)	-
no	3.33 (1/30)	-	10.00 (3/30)	13.33 (4/30)	26.67 (8/30)	16.67 (5/30)	-

**2016 (*n*=199)**							

**Household occupancy rate**							
1-3	20.83 (10/48)	6.25 (3/48)	20.83 (10/48)	18.75 (9/48)	8.33 (4/48)	2.08 (1/48)	0 (0/48)
3-5	20.00 (15/75)	5.33 (4/75)	17.33 (13/75)	14.67 (11/75)	6.67 (5/75)	5.33 (4/75)	1.33 (1/75)
>5	27.63 (21/76)	6.58 (5/76)	39.47 (30/76)	30.26 (23/76)	6.58 (5/76)	1.32 (1/76)	3.95 (3/76)
**Range of monthly income per capita** ^**1**^							
0-44	24.53 (26/106)	5.66 (6/106)	30.19 (32/106)	27.36 (29/106)	6.60 (7/106)	3.77 (4/106)	1.89 (2/106)
45-88	27.45 (14/51)	1.96 (1/51)	23.53 (12/51)	21.57 (11/51)	9.80 (5/51)	3.92 (2/51)	3.92 (2/51)
>88	7.14 (3/42)	11.90 (5/42)	21.43 (9/42)	7.14 (3/42)	4.76 (2/42)	0 (0/42)	0 (0/42)
**Presence of toilet**							
yes	20.25 (32/158)	12.20 (5/41)	21.52 (34/158)	18.99 (30/158)	5.70 (9/158)	3.80 (6/158)	2.53 (4/158)
no	34.15 (14/41)	4.43 (7/158)	46.34 (19/41)	31.71 (13/41)	12.20 (5/41)	0 (0/41)	0 (0/41)

Household occupancy rate: number of residents/household. ^1^Range of monthly income per capita, considering the dollar USD$ 2.27, quoted in July 2014.

**Table 2:** Prevalence of intestinal parasitic infections in relation to drinking water quality, classified as satisfactory or unsatisfactory, in the years 2014, 2015, and 2016 in the rural communities studied in Settlement 25 de Maio, Ceará State, Brazil.

	**2014**			**2015**			**2016**		
	**Water Quality** ^**∗**^ ** (*n*=151)**			**Water Quality** ^**∗**^ ** (*n*=184)**			**Water Quality** ^**∗**^ ** (*n*=199)**		
**Intestinal** ** Parasitoses**	**Satisfactory** ** (**%**)**	**Unsatisfactory ** **(**%**)**	***p*- value**	**Satisfactory (**%**)**	**Unsatisfactory (**%**)**	***p*- value**	**Satisfactory (**%**)**	**Unsatisfactory (**%**)**	***p*- value**

*Giardia intestinalis*	6/68 (8.8%)	3/83 (3.6%)	0.300	28/131 (21.4%)	9/53 (17.0%)	0.594	6/57 (10.5%)	8/141(5.7%)	0.463
*Iodamoeba butschlii*	_	_	_	_	_	_	7/57 (12.3%)	4/141 (2.8%)	0.063
*Entamoebahistolytica/dispar*	6/68 (8.8%)	3/83 (3.6%)	0.300	11/131 (8.4%)	2/53 (3.8%)	0.353	15/57 (26.3%)	28/141 (19.9%)	0.528
*Entamoeba coli*	7/68 (10.3%)	1/83 (1.2%)	0.022	19/131 (14.5%)	8/53 (15.1%)	1	17/57 (29.8%)	36/141 (25.5%)	0.688
*Endolimax nana*	_	_	_	7/131 (5.3%)	1/53 (1.8%)	0.441	15/57 (26.3%)	31/141 (22.0%)	0.694
*Ascaris lumbricoides*	24/68 (35.3%)	10/83 (12,1%)	0.001	38/131 (29.0%)	17/53 (32.1%)	0.723	4/57 (7.0%)	2/141 (1.4%)	0.111

^*∗*^The classification of water quality as satisfactory or unsatisfactory followed the parameters of water quality presented in Ordinance 2914 (December 12, 2011) of Brazil's Ministry of Health.

**Table 3:** Prevalence of intestinal parasitic infection in relation to the use of household-based water filters in 2016 in rural communities studied in Settlement 25 de Maio, Ceará State, Brazil.

**Household-based water filter**					
**Intestinal Parasites**	**Type 0 (**%**)**	**Type 1 (**%**)**	**Type 2 (**%**)**	**Type 3 (**%**)**	***p*-value**

*Giardia intestinalis*	5/66 (7.6)	4/48 (8.3)	1/24 (4.2)	4/61 (6.6)	0.924
*Entamoeba histolytica*	16/66 (24.2)	6/48 (12.5)	10/24 (41.7)	11/61 (18.0)	0.032
*Entamoeba coli*	18/66 (27.3)	14/48 (29.2)	11/24 (45.8)	10/61 (16.4)	0.046
*Endolimax nana*	15/66 (22.7)	10/48 (20.8)	7/24 (29.2)	14/61 (23.0)	0.886
*Iodamoeba butschlii*	15/66 (22.7)	2/48 (4.2)	2/24 (8.3)	3/61 (4.9)	0.818
*Ascaris lumbricoides*	1/66 (1.5)	3/48 (6.3)	0/24 (0.0)	2/61(3.3)	0.393

Types: 0: ceramic candle filter (hallow); 1: ceramic candle filter (with coal); 2: existing ceramic candle filter (unknown interior); 3: not using a household-based water filter.
